# Oxidative Stress in Fibromyalgia: From Pathology to Treatment

**DOI:** 10.1155/2022/1582432

**Published:** 2022-10-05

**Authors:** Chanika Assavarittirong, Włodzimierz Samborski, Bogna Grygiel-Górniak

**Affiliations:** Department of Rheumatology, Rehabilitation and Internal Diseases, Poznan University of Medical Sciences, Poznan, Poland

## Abstract

Fibromyalgia (FM) is characterized by chronic widespread musculoskeletal pain associated with sleep problems, fatigue, depression, and anxiety. The persistence of pain, impairment of cognitive function, and negative impact on the psychological state have caused a detrimental effect on the patients' quality of life. However, to date, the treatment and mechanisms of this disease are yet to be established. Oxidative stress might play a critical role in FM pathophysiology. Increased levels of prooxidative factors such as nitric oxide, lipid peroxidation, and mitophagy can cause pain sensitization in fibromyalgia. Numerous studies have supported the hypothesis of beneficial antioxidative effects in FM. Due to the lack of effective therapy for fibromyalgia, many treatments are sought to reduce pain and fatigue and improve patients' quality of life. This manuscript discusses the impact of various antioxidative procedures that can diminish fibromyalgia symptoms, such as hyperbaric oxygen therapy, modification of dietary habits, and physical activity.

## 1. Introduction

Fibromyalgia was initially classified by the American College of Rheumatology in 1990 as “fibrositis syndrome” characterized by chronic widespread musculoskeletal pain associated with sleep problems, fatigue, depression, and/or anxiety which impact daily cognitive functioning [1, 2]. Despite the unclear pathophysiological mechanism of the disorder, numerous studies attempted to investigate the underlying cause and mechanisms implicated in this disease, such as genetic predisposition; metabolic, biochemical, and immunological factors; correlation with psychological disorders; and dysfunction in pain modulating pathways [3].

The persistence of pain, impairment of cognitive function, and negative impact on the psychological state of fibromyalgia have caused a detrimental effect on the patients' quality of life (QoL). FM patients are characterized by significantly lower mental and physical health scores than the general population and other patients with chronic illnesses [4]. Since in fibromyalgia, poor health-related quality of life index and low socioeconomic impact are observed; finding an effective treatment for this disease could potentially improve patients' QoL, including mental and physical wellbeing [5–8]. Hence, this review intends to discuss the therapeutic methods to improve FM patients' QoL by exploring oxidative stress' pathogenesis and treatment principles.

The current fibromyalgia management comprises patient education, psychotherapy, exercise program, and pharmacotherapy [9, 10]. However, to date, the treatment of this disorder raises many clinical questions and is a field of scientific interest. Recent data showed that oxidative stress might play a critical role in this disease [11–14]. Thus, the approach to estimating anti- and prooxidative markers may help manage this disease more effectively. Recent studies show new aspects of antioxidative treatment in fibromyalgia patients. They describe hyperbaric oxygen therapy, aerobic exercises, and dietary modifications, which help achieve better treatment effects [11–14]. While the effective management of fibromyalgia is still unknown, the benefits of antioxidative therapy are being actively investigated. This review discusses the pathogenetic role of oxidative stress in fibromyalgia development and the beneficial aspects of antioxidants in treating this disease.

## 2. Pathophysiology of Fibromyalgia

**Figure 1:**
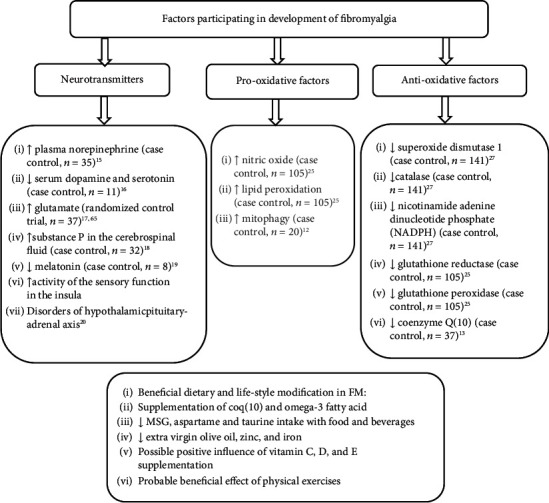
Pro- and antioxidative mechanism of FM and its lifestyle preventions.

Biochemical mechanisms in fibromyalgia are widely discussed in the literature. Cumulative evidence points at alterations in neurotransmitter systems in fibromyalgia, which influence pain perception, fatigue, sleep disturbances, and depressive as well as anxiety-related symptoms (Figure 1). Higher levels of plasma norepinephrine have been detected in FM patients [15]. In contrast, dopamine and serotonin levels were reduced compared to the healthy population, which could underline the diffuse pain sensation and discomfort [16]. On the other hand, glutamate causes higher sensitization of FM pain [17].

In the pathogenesis of fibromyalgia, crucial role plays neuropeptide substance P. Its concentration is threefold higher in the cerebrospinal fluid (CSF) than in healthy subjects. Substance P participates in excessive pain modulating and potentiating fibromyalgia symptoms [18]. This neuropeptide is stored in the vesicles of the presynaptic terminal axon and is released along with glutamate into the synaptic cleft during the nociceptive transmission.

Since many patients suffer from sleep problems, the melatonin level was also suspected of playing a role in the pathogenesis of fibromyalgia. It was proved that the low melatonin nighttime might intensify the nocturnal pain sensation [19]. Magnetic resonance imaging (MRI) of the brain discovered an increased activity of the sensory function in the insula during pain episodes [20]. Insula plays a role in sensory stimuli integration and processing. Its increased activity is associated with pain during symptom exacerbation [20].

Besides neurological background, endocrinological disorders were also present, such as the improper function of the hypothalamic-pituitary-adrenal axis, synthesizing the endorphin-releasing hormone, ACTH, and cortisol fibromyalgia [21]. In FM, hypofunctioning of the hypothalamic-pituitary-adrenal axis is observed, which results in adrenal insufficiency [22]. This insufficiency can potentially explain the symptoms of chronic fatigue, poor exercise ability, and reduced muscle performance in FM patients [21].

One of the crucial factors in the pathogenesis of fibromyalgia is oxidative stress. Recent data have shown a correlation between prooxidative processes and pain sensitization in FM subjects. In particular, the coenzyme Q10 (CoQ10) level is reduced and contributes to mitochondrial dysfunction. As a result, decreased potential of mitochondrial membrane, increased superoxide activity, and excessive synthesis of lipid peroxidation products are observed [11–13]. Eventually, cell mitophagy occurs, and autophagy is detected in blood mononuclear cells and plasma from patients with fibromyalgia [14]. In a physiological condition, autophagy could be beneficial as it eliminates dysfunctional cellular organelles. However, excessive autophagy in fibromyalgia can promote cell injury and pose higher oxidative stress risks [12].

Prooxidative processes are also observed in brain tissue. Due to the high proportion of lipids, the central nervous system is most vulnerable to free radical damage [3, 23]. Lipid peroxidation reactions increase the synthesis of oxidative stress markers such as hydroperoxides products and aldehydes, such as malonaldehyde and 4-hydroxynonenal [24]. It has been demonstrated that the levels of lipid peroxidation products positively correlate with the FM severity evaluated by the Fibromyalgia Impact Questionnaire-Revised (FIQR) [25, 26].

## 3. Anti- and Prooxidative Marker Activity in Fibromyalgia

**Figure 2:**
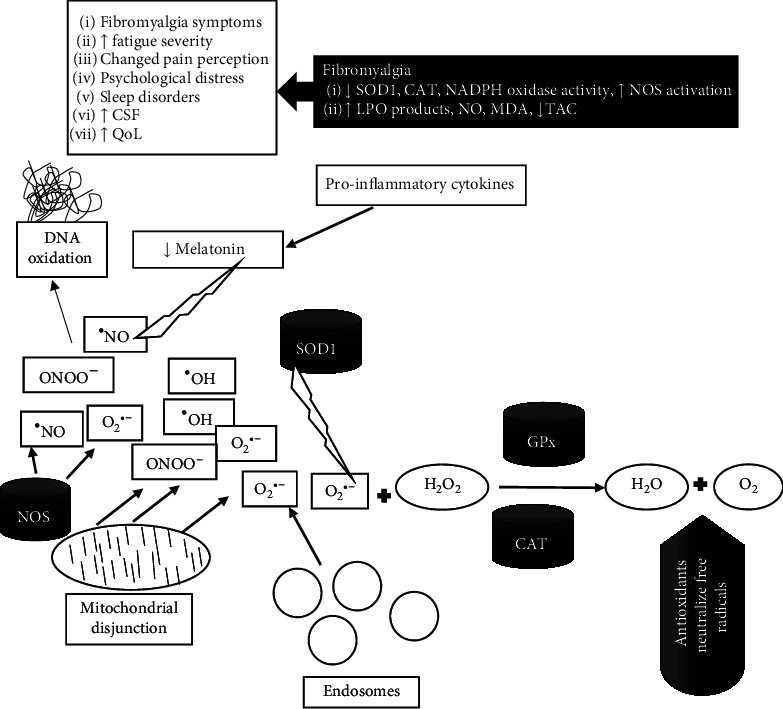
Molecular and oxidative processes in fibromyalgia. SOD1: superoxide dismutase 1; CAT: catalase; GPx: glutathione peroxidase; NADPH: nicotinamide adenine dinucleotide phosphate; O_2_^•−^: superoxide anion; NO: nitric oxide; ONOO^−^: peroxynitrite; MDA: malondialdehyde; LPO: lipid peroxidation; NOS: nitric oxide synthase; TAC: total antioxidant capacity; CSF: chronic fatigue syndrome; QoL: quality of life.

Many data show an imbalance between oxidants and antioxidants in FM patients (Figure 2). Prooxidative processes in FM patients are associated with specific gene variants, which participate in oxidative balance. For example, reduced functioning of superoxide dismutase 1 (SOD), catalase (CAT), and nicotinamide adenine dinucleotide phosphate (NADPH) oxidase correlates with pain and fatigue severity (assessed by the FIQR) [27]. SOD rapidly and specifically reduces superoxide radicals to hydrogen peroxide (H_2_O_2_), while H_2_O_2_ is decomposed to water by CAT and glutathione peroxidase (GPx) [28]. Moreover, low antioxidative enzyme activity (CAT, SOD, glutathione reductase (GR), and glutathione peroxidase (GPx) levels) and increased nitric oxide (NO) and MDA (a marker of lipid peroxidation (LPO)) in red blood cell lysate are observed in FM subjects. The antioxidative enzymes (Cat, GR, and GPx) are negatively associated with the severity of the symptoms evaluated by the FIQR as overall functioning, pain, psychological distress, and sleep. Conversely, LPO levels show a significant positive correlation with FIQR [25].

Another important marker of prooxidant in FM, which is related to pain sensation, is serum nitrite level [25]. An elevated marker level was found in experimental studies, revealing that NO is a crucial neurotransmitter involved in the spinal pain pathway. The sensitization of those pathways is related to nitric oxide synthase (NOS) activation, which causes subsequent NO elevation [29, 30].

Besides the mechanisms described above, the ability to remove free radicals is significantly decreased in FM due to mitochondrial dysfunction [11–13]. Consequently, the susceptibility to oxidative stress and mitochondrial dysfunction can increase the synthesis of free radicals and mediate inflammatory cytokines that may play a role in pain generation [31]. Thus, treatments involving antioxidant effects may help improve the harmful FM manifestations.

The beneficial effects of decreased prooxidants and increased antioxidative capacity arouse many interests in scientists. The study of La Rubia et al. confirmed that the lower antioxidant enzyme activities might lead to oxidative stress through the oxidation of DNA and protein carbonyl content (general indicator marker of protein oxidation), which may affect the health status of FM patients [32]. Moreover, diminished total antioxidant capacity (TAC) in FM women was compared to the control group [33, 34].

Since dysfunction of the central nervous system is involved in the physiopathology of fibromyalgia, many studies analyze the substances participating in brain regulation. Melatonin is a hormone that improves sleep quality and increases the pain threshold. In an animal model, melatonin supplementation was shown to prevent the deterioration of the myotubes in gastrocnemius muscles in rats' riserpine-induced myalgia (RIM). In addition, increased mitochondrial markers and CoQ10 in gastrocnemius muscles were observed. This study shows that the use of melatonin supplementation not only has antioxidative properties but also can diminish musculoskeletal pain [35].

The clinical studies also confirm the anti-inflammatory activity of melatonin and its positive influence on neuropathic and chronic pain [36–39]. Recent data show that melatonin reduces oxidative stress by its potential radical scavenging properties [38]. This hormone has a beneficial influence on chronic fatigue syndrome (CFS)—a component of fibromyalgia. CFS is characterized by disabling physical and mental fatigue linked to postexertional malaise and does not improve with rest. It seriously interferes with work activity and daily life tasks [39]. Melatonin diminishes fatigue during the day and reveals anxiolytic properties [39, 40]. Unfortunately, FM patients have low melatonin secretion during the hours of darkness than healthy subjects. Insufficient melatonin synthesis may contribute to poor sleep at night, fatigue during the day, and changed pain perception [41]. Thus, melatonin administration in fibromyalgia can reduce pain and improve sleep quality, improving the overall quality of life [42].

SOD1 is responsible for converting the toxic superoxide anion (O_2_^•−^) into oxygen (O_2_) and hydrogen peroxide (H_2_O_2_). Later, H_2_O_2_ is decomposed into the water by CAT and GPx. Nitric oxide (NO) is produced by nitric oxide synthase (NOS). Peroxynitrite (ONOO^−^) is generated by nitric oxide (NO) and superoxide anion (O_2_˙^−^) and influences brain processes. NO is a signaling free radical molecule, functioning as a vascular smooth muscle relaxant, neurotransmitter, and immune regulator, which causes sensitization of the spinal pain pathway in fibromyalgia. The ability to remove free radicals is significantly decreased in FM due to mitochondrial dysfunction. Insufficient antioxidant enzyme activities and diminished total antioxidant capacity (TAC) in FM lead to oxidative stress through the oxidation of DNA. Low melatonin secretion during the night is observed in FM patients caused by reactive oxygen species. Melatonin has potential radical scavenging activity and can reduce oxidative stress. Decreased melatonin secretion causes neuropathic pain and stimulates the development of chronic fatigue syndrome (CFS).

## 4. An Antioxidative Mechanism in Fibromyalgia Treatment

Treatment using the antioxidative mechanisms has improved the overall FM symptoms. Numerous studies have supported the hypothesis of beneficial antioxidants therapy for this disease through clinical therapeutics such as hyperbaric oxygen therapy [43], aerobic exercises (yoga and Tai Chi) [44, 45], and antioxidant supplementation (CoQ10 and vitamins D and E) [46–49].

## 5. Hyperbaric Oxygen Therapy: Additive Management in Fibromyalgia

A promising mechanism of hyperbaric oxygen therapy is its ability to reduce oxidative stress in a local hypoxic condition. This therapy deactivates caspase 3 and caspase 9 and increases apoptosis regulatory gene Bcl-2 expression [43, 50]. This suggests that hyperbaric oxygen therapy may increase cellular oxygen availability, reduce mitochondrial-induced apoptosis, and preserve mitochondrial function. Therefore, the reduced inflammatory cytokines seem to cause a global improvement in functioning and pain reduction in fibromyalgia patients (Table 1).

In animal models, hyperbaric oxygen therapy reduces lipid peroxidation and decreases the prooxidative processes [51] . The controversy regarding this therapy concerns reactive oxygen species (ROS) generation in physiological conditions. Therefore, healthy subjects might increase ROS synthesis. However, hyperbaric oxygen therapy has shown positive effects in clinical trials of fibromyalgia treatment, such as improved global functioning, pain reduction, psychological relief, and normalization of brain activity [52–57].

## 6. Nutritional Antioxidants in the Treatment of Fibromyalgia

As previously mentioned, reduction of CoQ correlates with the worsening of fibromyalgia symptoms (10). The low level of CoQ10 contributes to mitochondrial dysfunction and the generation of ROS and lipid peroxidation [11–13]. The effect of CoQ10 dietary supplementation in patients with fibromyalgia was analyzed in randomized controlled studies and case reports [46, 47]. It was proved that combined supplementation of CoQ10, vitamin D, alpha-lipoic acid, magnesium, and tryptophan causes a significant reduction of pain after one month of treatment and noticeable improvement in QoL [47]. Furthermore, CoQ10 supplementation effectively alleviates pain in fibromyalgia patients already treated with pregabalin [46]. The study of Cordero et al. (RCT) revealed that the combined therapy of CoQ10 and pregabalin significantly reduced pain level and anxiety compared to pregabalin monotherapy [46]. Beneficial effect of CoQ10 supplementation was also observed in a case report and resulted in reduced oxidative stress markers, improved mitochondrial functions, and diminished clinical symptoms [46]. Thus, CoQ10 addition to standard fibromyalgia therapy may be beneficial in improving the signs by restoring mitochondrial function and reducing oxidative stress.

Besides CoQ10 supplementation, dietary antioxidant intake (including vitamins A, C, D, and E) can be helpful in fibromyalgia treatment. However, ambiguous results were observed. A randomized controlled trial of Warner and Arnspiger presented no significant improvement after 50,000 IU of vitamin D supplementation once per week for three months [49]. On the other hand, the study by Wepner et al. has found decreased sensitization of pain measured visual analogue scale (VAS) and diminished morning fatigue in the Fibromyalgia Impact Questionnaire (FIQ) caused by adding 1200 IU or 2400 IU vitamin D daily for 25 weeks [48].

The oxidative stress parameters and fibromyalgia symptoms were reduced after supplementing antioxidative vitamins. The case series of vitamin A supplementation reported withdrawal or decreased fibromyalgia symptoms (such as generalized musculoskeletal pain, fatigue, and depressed mood) [58]. Moreover, a controlled trial found that supplementing vitamins C (200 mg) and E (200 mg) daily for 8 weeks resulted in pain reduction [59]. On the contrary, the study of Nazıroğlu et al. did not confirm this effect. In a controlled clinical pilot study, the authors did not observe significant changes after 12 weeks of daily supplementation of vitamins C (500 mg) and E (150 mg) [60]. The inconsistent results could be attributed to the different dosages of vitamin supplementation, which may influence pain modulation. Moreover, the exact mechanism of vitamin effect on fibromyalgia symptoms is not fully discovered and will require further investigations.

One of the valuable dietary products with antioxidant properties is extra virgin olive oil. It contains various natural polyphenols, demonstrating high antioxidative effectiveness in reducing ROS [60]. It was proved that consuming 50 mL of olive oil daily for three weeks improves FIQ score, physical ability, and mental health in 23 fibromyalgia patients [61]. Furthermore, olive oil consumption caused decreased cardiovascular risks (improved lipid profile, inflammatory markers, and thrombosis-related parameters) in 15 females with fibromyalgia [61]. Future studies in bigger groups of patients can confirm the hypothesis that olive oil benefits in discussed disease.

The antioxidative essential trace element that positively influences fibromyalgia symptoms is zinc. The primary dietary sources of this microelement are oysters and red meats. Zinc is critical for the formation and activity of many enzymes and cells that play a role in maintaining an immunologic balance. This microelement is a cofactor of many antioxidant enzymes such as superoxide dismutase. Serum zinc concentration is significantly lower in chronic fatigue syndrome, related to oxidative stress [62]. This fact is supported by another clinical study, which shows substantially lower serum zinc levels (*p* < 0.001) in 45 female patients with fibromyalgia [32]. Similar observation is confirmed in other studies [63, 64]. Conversely, zinc deficiency was positively correlated with the severity of depression (a crucial clinical presentation in fibromyalgia) [65].

Selenium is the essential trace element of antioxidative properties, influencing neurotransmission. Unfortunately, selenium level is reduced in FM patients [66, 67]. Selenium is a cofactor of the glutathione peroxidase (GSH-Px) enzyme [68]. It is a regulator of the physiological functions of the nervous system, such as signal transduction and development [47]. Moreover, it is a crucial component of selenoproteins involved in antioxidant defenses [68]. This trace element shows neuroprotective properties in peripheral pain modulation through the inhibition of apoptosis and regulation of the transient receptor potential (TRP) melastatin 2 (TRPM2) and vanilloid 1 (TRPV1) channels [69]. The study by Yüksel et al. in an animal model explored the role of selenium in FM-induced rats. After selenium supplementation, reduced pain intensity, ROS level, and lipid peroxidation were observed [69]. Selenium reveals neuroprotective properties in the nigrostriatal pathway. Thus, the dopamine pathway is thought to be selenium-dependent [70]. Selenoproteins are necessary for the development of parvalbumin- (PV-) interneurons, a class of GABAergic neurons involved in synchronizing neural activity [66]. The dysfunctional activity of PV-interneuron networks is involved in hyperalgesia in fibromyalgia, in which pain information processing is amplified in the central nervous system [67]. Thus, selenium supplementation in deficient FM patients can improve clinical symptoms and benefit the quality of life.

Another nonenzymatic dietary antioxidant is iron. Patients with fibromyalgia have significantly reduced serum ferritin levels compared to healthy controls (*p* = 0.003) [71]. A study performed by Pamuk et al. has found an increased risk of fibromyalgia prevalence in females with iron deficiency anemia [72]. A similar data published by Yao et al. confirmed the high risks of FM incidence in iron-deficient individuals [73]. Clinical, placebo-controlled trial study investigated the effect of ferric carboxymaltose on 80 females with FM. After 42 days of iron supplementation, the experimental group improved the FIQR score, enhanced in brief pain inventory (BPI) score, and reduced fatigue [74].

Recent studies also show the beneficial outcome of folic acid supplementation in FM patients. Folic acid has antioxidative properties and improves the function of the immune system and pain sensation [75]. The prooxidative processes induce activation of mast cells, and systemic macrocytosis is observed in fibromyalgia [76]. Mast cells are located near the nerve fibers, allowing them to migrate for modulating nociception and neural activity (they communicate with microglia) [77]. This mechanism maintains the pain sensation even after the original stimulus is over [78]. In an animal study by Fusco et al., the effects of melatonin and folic acid supplementation were also investigated in the RIM rat model. Concomitant treatment of melatonin and folic acid supplementation restores the enzymatic activity of superoxide dismutase and significantly reduces lipid peroxidation in the RIM rats [79]. Such supplementation decreased an elevated in fibromyalgia pain sensibility and depression-like behavior with more efficacy than single administration [79]. However, further clinical studies should give the answer if such supplementation is effective in patients with fibromyalgia.

Since many antioxidative nutrients may influence the FM symptoms, future studies should focus on their supplementation in large groups for a longer time. Precise estimation of the prooxidative effect of nutrients in fibromyalgia may improve the management of this disease.

## 7. Prooxidative Nutritional Factors in the Development of Fibromyalgia

Another nutrient that may increase fibromyalgia symptoms is the excessive intake of copper. Copper is present in various dietary products such as shellfish, seeds, nuts, and organ meats. The level of serum copper and ceruloplasmin (a copper-carrying protein) was elevated in fibromyalgia patients (*n* = 45) when compared to healthy controls (*p* < 0.001 and *p* < 0.001, respectively) [32]. Similar data were presented by La Rubia et al., Abdulnasser et al., and Shukla et al., who observed that serum copper, manganese, and aluminum concentration were significantly higher in fibromyalgia patients than in healthy controls. Moreover, the higher concentration of these metals correlated with high FIQR and manifested as increased tender points and pain sensation in FM subjects [32, 63, 80]. Thus, copper and ceruloplasmin may play a role in contributing to the oxidative stress pathogenesis of fibromyalgia. However, there is still inadequate evidence to support the hypothesis that copper can increase fibromyalgia risk. Therefore, accurate assessment of copper intake and its influence on fibromyalgia course should be the subject of scientific interest.

Some data show that some dietary components might potentiate the risk of fibromyalgia or exacerbate its symptoms. One of them is excitotoxin, a common nutritional additive. It increases fibromyalgia severity, mainly central pain sensitization [81]. Excitotoxins include monosodium glutamate (MSG) and aspartame, exacerbating fibromyalgia symptoms. Their high intake increases excitatory neurotransmission, generates ROS, and contributes to CNS oxidative stress [82]. A study by Holton et al. (randomized controlled trial) found that dietary glutamate significantly increases the severity of the symptoms of FM patients (assessed by worsening FIQR score, *p* < 0.03) [83]. Moreover, glutamate delivered with diet seems to correlate with ROS synthesis in the CNS and reduce the gut microbiome. Thus, diets containing excitotoxins such as glutamate or aspartame should be avoided in FM subjects.

Recent studies assessed the influence of reduced MSG and aspartame in the diet in fibromyalgia patients. The case studies found potential benefits in stopping dietary MSG and aspartame in five fibromyalgia patients [44, 84]. However, a study by Vellisca and Latorre showed no significant difference in the pain severity in 36 female FM patients following the discontinuation of dietary MSG and aspartame for three months [85]. Nevertheless, omega-3 polyunsaturated fatty acid (omega-3 PUFA) supplementation can prevent excess excitatory neurotransmission caused by excitotoxins [86]. Thus, patients who can not stop consuming dietary products which contain this MSG or aspartame should add omega-3 PUFA (present in olive oils) to the diet balancing the harmful activity of excitotoxins.

Taurine, an amino acid often added to energy drinks, is found in meat and seafood and could reveal a prooxidative action in fibromyalgia [87]. An increased level of this amino acid has been found in the serum of fibromyalgia patients, but it was not significantly associated with clinical manifestations determined by the FIQR [88]. It has been hypothesized that taurine could play a role in the hyperexcitability state in chronic pain by mediating calcium influx into excitable tissues. Taurine also is released with substance P during inflammation of painful arthritis in animal models. A study performed by Larson et al. presented a significant relationship between the CSF level of taurine and the tender point index (*p* < 0.001) in fibromyalgia patients. Therefore, dietary restriction of taurine could be beneficial to FM patients [89]. However, no study has analyzed the reduced or eliminated taurine intake on fibromyalgia symptoms until now.

## 8. Physical Activity

Various outcomes have been observed in an aerobic exercise intervention. Aerobic exercises may include walking, cycling, swimming, dance, or rhythmic movements [76]. However, some reports underline these exercises' side effects and increased fibromyalgia pain [90]. On the other hand, FM subjects who underwent 12 weeks of Tai Chi and aerobic exercise practices showed significantly reduced FIQR scores and benefits with reduced anxiety, pain, and global assessments (randomized trial) [45]. Similar results were observed in the telerehabilitation program on aerobic exercise for Spanish fibromyalgia patients [91]. However, the oxidative stress markers were not assessed in this study.

Another activity tested in FM patients is the whole-body vibration exercise. Such activity increases superoxide dismutase (*p* = 0.019) and reduces catalase activity (*p* = 0.005). Moreover, it decreases iron reduction capacity (*p* < 0.001) and diminishes the concentration of thiobarbituric acid reactive substances (*p* < 0.001) [92]. Thus, physical exercises' role in antioxidative activity requires further investigations on their benefits in patients with fibromyalgia.

## 9. Pharmacotherapy

Currently, several medications are utilized in the management of FM. These include antidepressants such as low-dose amitriptyline and serotonin and norepinephrine reuptake inhibitors (SNRIs), for example, duloxetine, mirtazapine, and venlafaxine. The analgesics such as tramadol and possibly other weak opioids and acetaminophen can be used in this disease. The mentioned antidepressants increase the neurotransmitters such as serotonin and norepinephrine in FM patients. The increased neurotransmitters' concentration in the synaptic space improves sleep and reduces pain and fatigue. However, the antidepressants do not affect the tenderness of tender points [93]. Pain reduction has been observed with pramipexole, pregabalin, and gabapentin. However, the efficacy in overall improvements of symptoms of pregabalin may not be as comparable to that of SNRIs and may induce more somatic symptoms seen in FM [94].

## 10. Conclusions

Despite the small sample size groups in many studies, the randomized clinical trials showed positive hyperbaric oxygen therapy results in reducing the oxidative markers in fibromyalgia. The data concerning the nutritional antioxidants are very promising. For example, dietary supplementation of CoQ10 positively influences FM symptoms. Inconsistent results were observed with vitamins C, D, and E supplementation as well as physical exercises. Reducing MSG, aspartame, and taurine intakes with food and beverages may help improve the overall symptoms of fibromyalgia. Extra virgin olive oil, zinc, and iron may also provide benefits in reducing ROS, followed by diminished cardiovascular risks in FM subjects. Future studies should give a reliable background of the underlying pro- and antioxidative mechanisms related to specific physical activity and dietary products' nutritional supplementation. Nevertheless, the supplementation of CoQ10 and omega-3 fatty acid to standard therapy can be helpful for patients with fibromyalgia. The oxidative stress in fibromyalgia may contribute to the pathogenesis of this rheumatic disease. However, further studies are needed to establish the mechanism of the disease. Finally, treatments involving antioxidative methods have shown promising results in improving various FM symptoms, including the QoL.

## Figures and Tables

**Table 1:** The effect of hyperbaric oxygen therapy on the fibromyalgia management.

Hyperbaric oxygen therapy in fibromyalgia management			
Type of study	*n*	Used methods	Effects
Observational clinical study [54]	*n* = 28	20- to 90-minute hyperbaric oxygen therapy sessions—repeated 10 to 20 times	(i) ↓Pain and anxiety after 10 sessions (ii) ↓Fatigue after 20 sessions

Cohort study [52]	*n* = 18	100% hyperbaric oxygen therapy used 5 days per week for 8 weeks	(i) ↑Overall global functioning (ii) ↓Anxiety and depression (iii) ↑Sleep quality (iv) Improvements retained after 3-month treatment follow-up Adverse effects: (i) Mild barometric injury, including mild middle-ear barotrauma associated with new-onset myopia (*n* = 3) (ii) Separate myopia development (*n* = 4)

Randomized controlled trial [53]	*n* = 49	Physical exercise group (*n* = 16) vs. low-pressure hyperbaric oxygen therapy group (*n* = 17) vs. control group (*n* = 16) Hyperbaric oxygen therapy: 100% oxygen with airbrakes at 1.45 ATA (90-minute sessions, 5 sessions per week, the total amount of sessions = 40)	(i) ↓Pain and fatigue in hyperbaric oxygen therapy intervention group (ii) ↑Pain threshold, physical performance, and endurance in hyperbaric oxygen therapy and physical exercise groups

Prospective crossover clinical trial [55]	*n* = 60	40 sessions of hyperbaric oxygen therapy (5 times per week, 90 minutes per session)	(i) ↓Tender points of pain (ii) ↑FIQR score (iii) ↓Psychological distress (iv) ↑QoL assessment scores (v) ↑The activity of Brodmann areas in the frontal lobe (vi) ↓Activity in the posterior region (SPECT imaging)

A randomized crossover controlled trial [56]	*n* = 30	Hyperbaric oxygen therapy group (90 minutes per session, five days per week, for a total of 60 sessions) vs. psychotherapy group	(i) ↓Pain sensation (ii) ↑QoL (iii) ↓PTSD symptoms (iv) Improved brain function (showed in SPECT imaging) (v) ↑Brain activity in the prefrontal cortex, orbital frontal cortex, and the subgenual area

Abbreviations: *n*: number of patients; ATA: atmosphere absolute; FIQR: Fibromyalgia Impact Questionnaire-Revised; QoL: quality of life; PTSD: posttraumatic stress disorder; SPECT: single-photon emission computerized tomography.
